# Supplementary material for: A tri-ionic anchor mechanism drives Ube2N-specific recruitment and K63-chain ubiquitination in TRIM ligases
Source: Nat Commun. 2019 Oct 3;10:4502. doi: 10.1038/s41467-019-12388-y (PMC6776665; doi:10.1038/s41467-019-12388-y)
Supplement: Supplementary file 1 — Supplementary Information [file 41467_2019_12388_MOESM1_ESM.pdf]

Supplementary Information

A tri-ionic anchor mechanism drives Ube2N-specific recruitment and K63-chain ubiquitination in TRIM ligases

Kiss *et al.*

This PDF includes:

- Supplementary Tables 1 and 2
- Supplementary Figures 1-13

Supplementary Table 1 Data collection and refinement statistics (molecular replacement)

	TRIM21-RING:Ube2N~ubiquitin (6S53)
**Data collection**	
Space group	P1
Cell dimensions	
a, *b*, *c* (Å)	49.75, 83.31, 86.75
α, β, γ (°)	89.90, 89.05, 88.70
Resolution (Å)	86.74 - 2.8 (2.9 - 2.8)
R_{merge}	0.03857 (0.3994)
$I / \sigma I$	12.83 (1.69)
Completeness (%)	95.74 (96.05)
Redundancy	1.8 (1.8)
**Refinement**	
Resolution (Å)	19.76 - 2.8 (2.9 - 2.8)
No. reflections	58804 (5933)
R_{work} / R_{free}	0.21/0.25
No. atoms	9476
Protein	9454
Ligand/ion	16
Water	6
B-factors	79.03
Protein	79.04
Ligand/ion	77.24
Water	59.79
R.m.s. deviations	
Bond lengths (Å)	0.009
Bond angles (°)	1.31

Data originated from one single crystal. Values in parentheses are for highest-resolution shell.

Supplementary Table 2 Primers used in this study.

Primer description	Primer sequence 5'-3'
TRIM21 E12A FWD	ATGTGGGCCGAGGTCACATGCCCTATCTGCCTG
TRIM21 E12A REV	CCTCGGCCCACATCATTGTCAAGCGTGCTGCTGA
TRIM21 E12R FWD	*ATGTGGCGCGAGGTCACATGCCCTATCTGCCTG*
TRIM21 E12R REV	CCTCGCGCCACATCATTGTCAAGCGTGCTGCTGA
TRIM21 E13A FWD	*GGGAGGCGGTCACATGCCCTATCTGCCTGGAC*
TRIM21 E13A REV	TGTGACCGCCTCCCACATCATTGTCAAGCGTGCT
TRIM21 E13R FWD	*GGGAGCGAGTCACATGCCCTATCTGCCTGGAC*
TRIM21 E13R REV	TGTGACTCGCTCCCACATCATTGTCAAGCGTGCT
TRIM21 D21R FWD	GCCTGCGGCCCTTCGTGGAGCCTGTGAG
TRIM21 D21R REV	GAAGGGCCGCAGGCAGATAGGGCATGTGACCTC
TRIM21 R55A FWD	CGCTGAGCGCACACAGGACAGACGCTGCC
TRIM21R55A REV	TCTGTCCTGTGTGCGCTCAGCGCTTTCTGCTCAAG
hTRIM5 E11R fwd	GTTAATGTAAAGCGCGAGGTGACCTGCCCCATCTG
hTRIM5 E11R rev	GGTCACCTCGCGCTTTACATTAACCAGGATTCC
hTRIM5 E12A fwd	AGGAGGCCGTGACCTGCCCCATCTGCCTG
hTRIM5 E12A rev	GGTCACGGCCTCCTTTACATTAACCAGGATTCCAGAAGCC
hTRIM5 E20R rev	CAGGAGGCGCAGGCAGATGGGGCAGGTC
hTRIM5 E20R fwd	TGCCTGCGCCTCCTGACACAACCCCTGAGCCTG
hTRIM5 R59A fwd	CCCTGTGTGCGCTATCAGTTACCAGCCTGAGAAC
hTRIM5 R59A rev	GGTAACTGATAGCGCACACAGGGCAGCTACTCTCTCC
Gibson fwd	TTAACGGCACACTTGACAATGGCTTCAGCAGCACGC
Gibson rev	TCGACTCTAGAGTCGCGGCCGCTCAATAGTCAGTGGATCCTTGTGATCCAATATTCAG
Ube2D1-D12R-fwd	GAGGATTCAGAAAGAATTGAGTcgcCTACAGCGCGATCCACCTGC
Ube2D1-D12R-rev	GCAGGTGGATCGCGCTGTAGgcgACTCAATTCTTTCTGAATCCTC
Ube2N-R14D-fwd	GCAGGATCATCAAGGAAACCCAGgatTTGCTGGCAGAACCAGTTCC
Ube2N-R14D-rev	GGAACTGGTTCTGCCAGCAAatcCTGGGTTTCCTTGATGATCCTGC
Ub-K11E-fwd	CTTCGTGAAGACCCTGACTGGTgaaACCATCACTCTCGAAGTGGAGCC
Ub-K11E-rev	GGCTCCACTTCGAGAGTGATGGTttcACCAGTCAGGGTCTTCACGAAG
TRIM21-RL-M10E/E12R-fwd	GCACGCTTGACAATGgaGTGGcgtGAGGTCACATGCCCTATCTGCC
TRIM21-RL-M10E/E12R-rev	GGCAGATAGGGCATGTGACCTCacgCCACtcCATTGTCAAGCGTGC
TRIM21-RL-M10E/E12A-fwd	CAGCACGCTTGACAATGgaGTGGgcgGAGGTCACATGCCCTATCTGCCTG
TRIM21-RL-M10E/E12A-rev	CAGGCAGATAGGGCATGTGACCTCcgcCCACtcCATTGTCAAGCGTGCTG
TRIM21-RL-M10E/E13A-fwd	GCACGCTTGACAATGgaGTGGGAGgcgGTCACATGCCCTATCTGCCTGGAC
TRIM21-RL-M10E/E13A-rev	GTCCAGGCAGATAGGGCATGTGACcgcCTCCCACtcCATTGTCAAGCGTGC

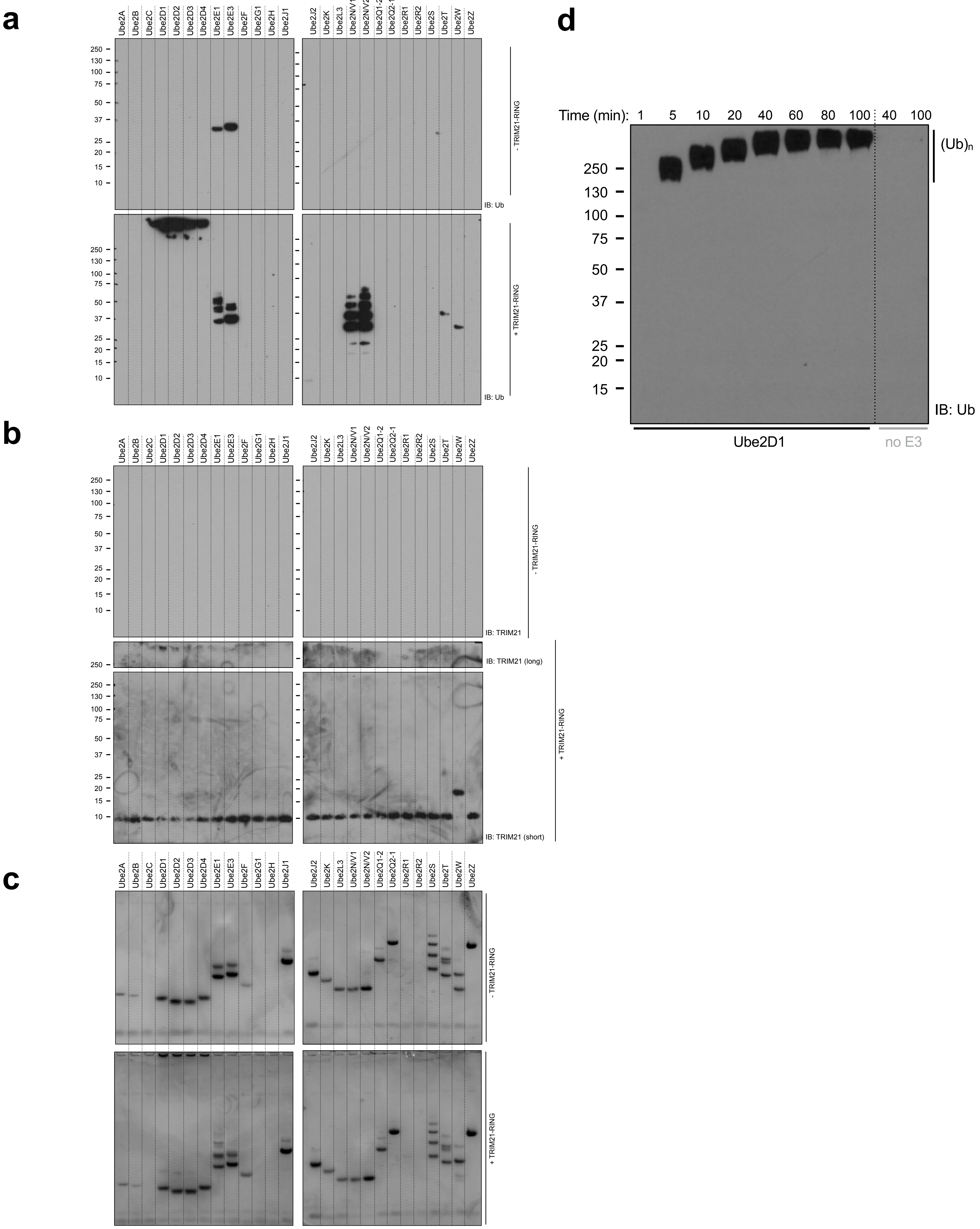

Supplementary Fig. 1 E2 screen. Full western blots and gels of the biochemical E2 screen (Boston Biochem, Cambridge, MA, USA) are shown. **a** Anti-Ub and **b** anti-T21-R western blots are shown. **c** The gels that were used for blotting and stained afterwards and are shown (without markers, as they were transferred to the membrane). The anti-T21-R western blot is shown for two different developing times, demonstrating that interaction of T21-R with Ube2D1 can result in the formation of anchored chains in addition to free ubiquitin chains. The anti-ubiquitin blot shows a band for Ube2T in presence of TRIM21. However, no difference could be detected in the gel. **d** Catalysis of Ube2D1 to confirm that the high molecular weight species for Ube2D1-4 represent ubiquitin chains. Source data are provided as a Source Data file.

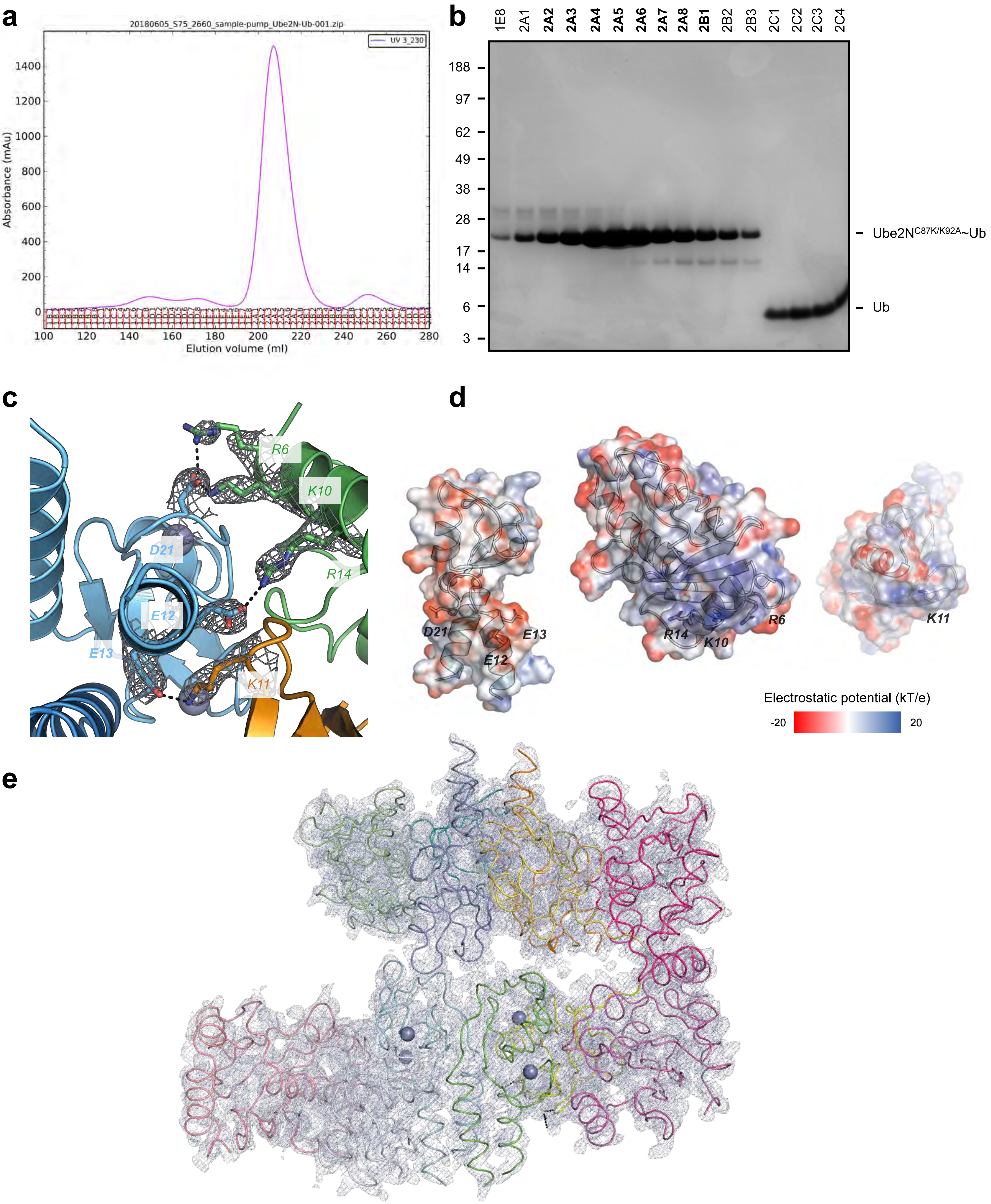

Supplementary Fig. 2 X-ray structure of TRIM21-RING in complex with Ube2N~Ub. **a** Gel filtration chromatogram and **b** LDS-gel of the purification of isopeptide-linked Ube2N$^{C87K/K92A}$~Ub, which was used for structural studies. **c** Anchor points motif close-up as in Fig. 2e, showing the 2F$_O$-F$_C$ density at 1.0 sigma for the residues involved. **d** Electrostatic potential surfaces from -20 (red) to +20 kV e^{-1} (blue). Electrostatic potential surfaces were generated with the APBS 2.1 tool in PyMol. PQR files were generated by PDB2PQR using the Amber force field. **e** Shown is the protein in the asymmetric unit (two full complexes) as cartoon and the 2F$_O$-F$_C$ density at 1.0 sigma.

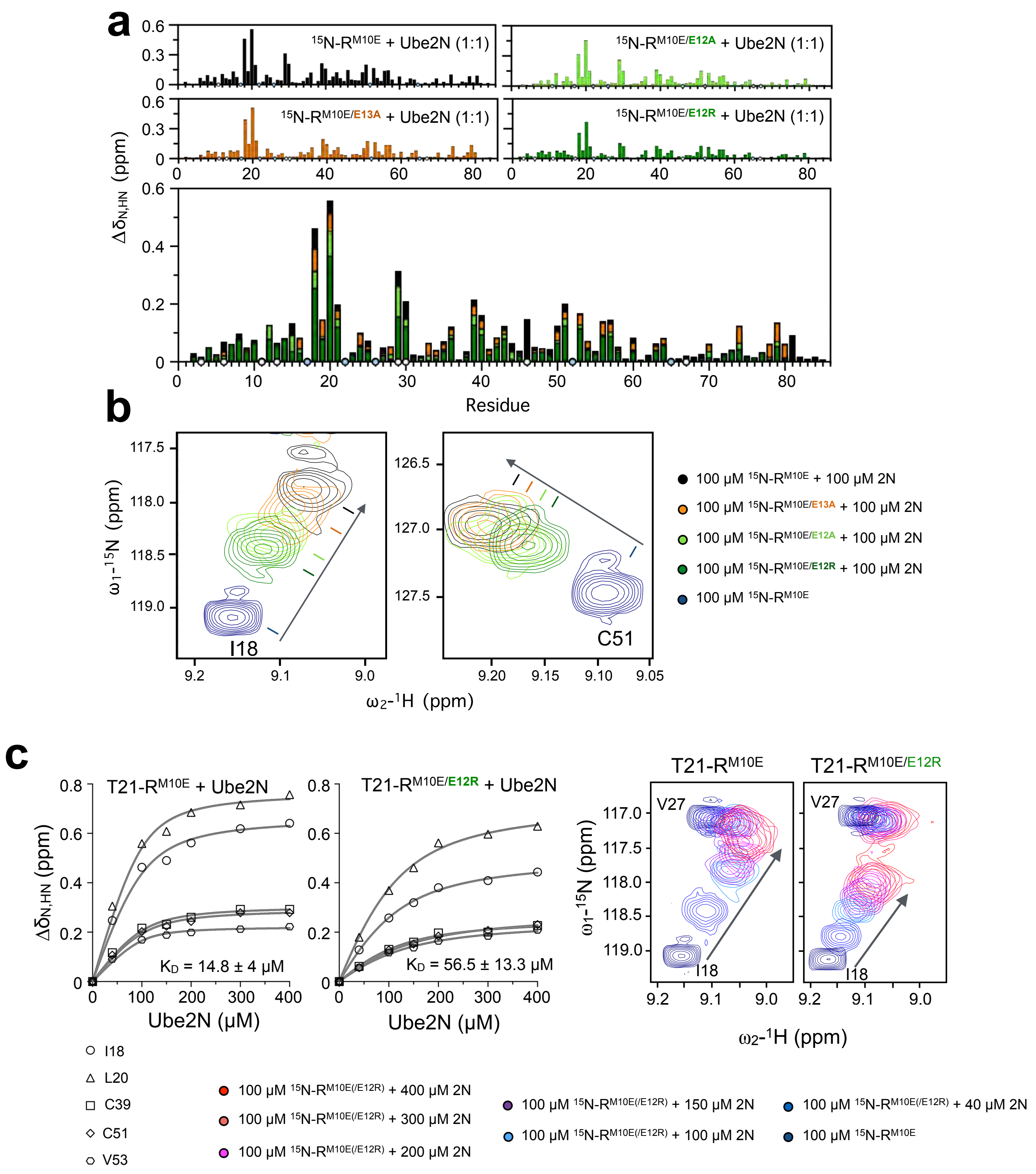

Supplementary Fig. 3 The effect of the tandem glutamates in binding Ube2N. **a** Histograms for the titrations of Ube2N into T21-R^{M10E}, showing CSP vs. sequence position. Blue circles indicate proline residues, white circles missing assignments. **b** Isolated peaks of selected amides (I18 and C51) of T21-R^{M10E} (abbreviated R^{M10E}) are shown in absence of titrant and different RING mutants in presence of 1 molar equivalent of Ube2N (abbreviated 2N). Full spectra are shown in Supplementary Fig. 4. **c** Dissociation constant fitting plots and peak of amide I18 are shown for T21-R^{M10E} and T21-R$^{M10E/E12R}$ titration with Ube2N. K_D values represent the mean ± s.d. of 5 different peaks that were fitted as described in Methods.

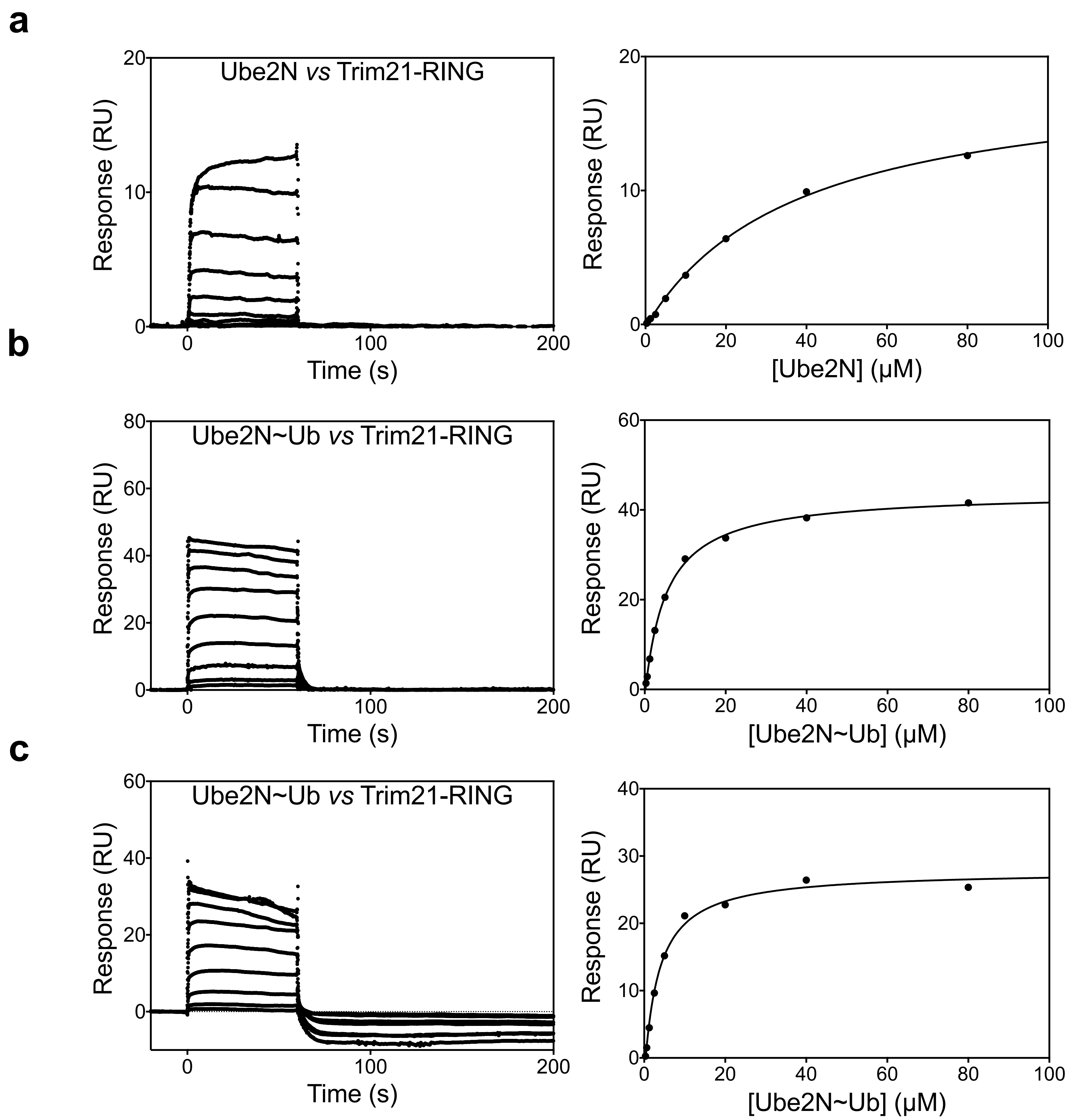

Supplementary Fig. 4 Surface plasmon resonance measurements. Replicate surface plasmon resonance data (sensograms and binding plots) for **a** Ube2N titration into GST-T21-R and **b**,**c** Ube2N~Ub.

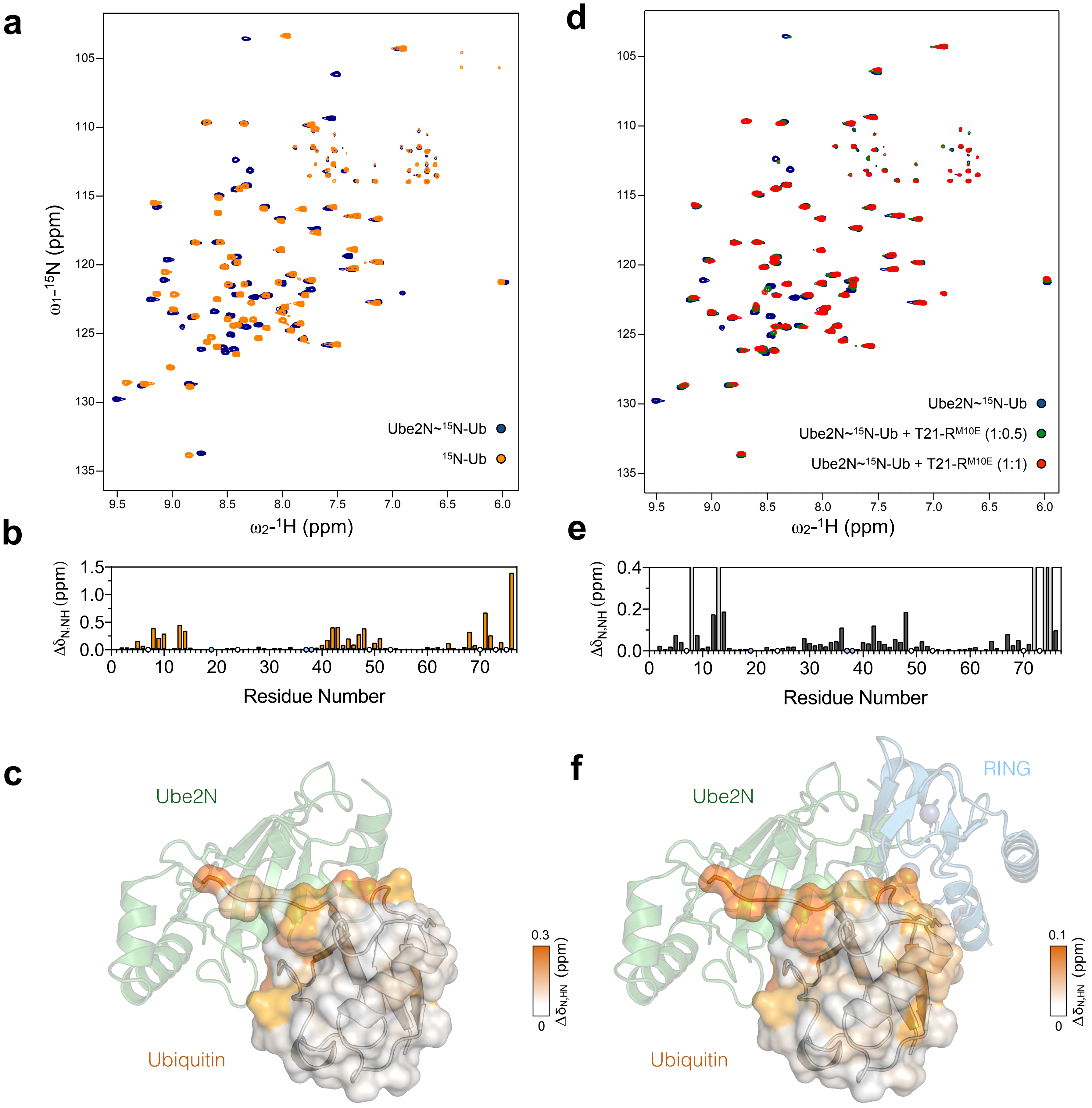

Supplementary Fig. 5 Formation of the closed Ube2N~Ub conformation in solution**.** Overlay of the ^{15}N-BEST-TROSY spectra of **a** free ^{15}N-ubiquitin (orange) and Ube2N~^{15}N-Ub (blue) and **d** the titration of T21-R^{M10E} into Ube2N~^{15}N-Ub (blue, free; green, half molar equivalent; red, one molar equivalent). **b**,**e** The CSPs are plotted against the residue number. In case of the titration, the CSP are calculated between the free and one molar equivalent added titrant. Blue circles indicate proline residues, white circles missing assignments. **c**,**f** The amide CSP between **c** ^{15}N-Ub and **f** Ube2N~^{15}N-Ub are mapped onto the structure in orange.

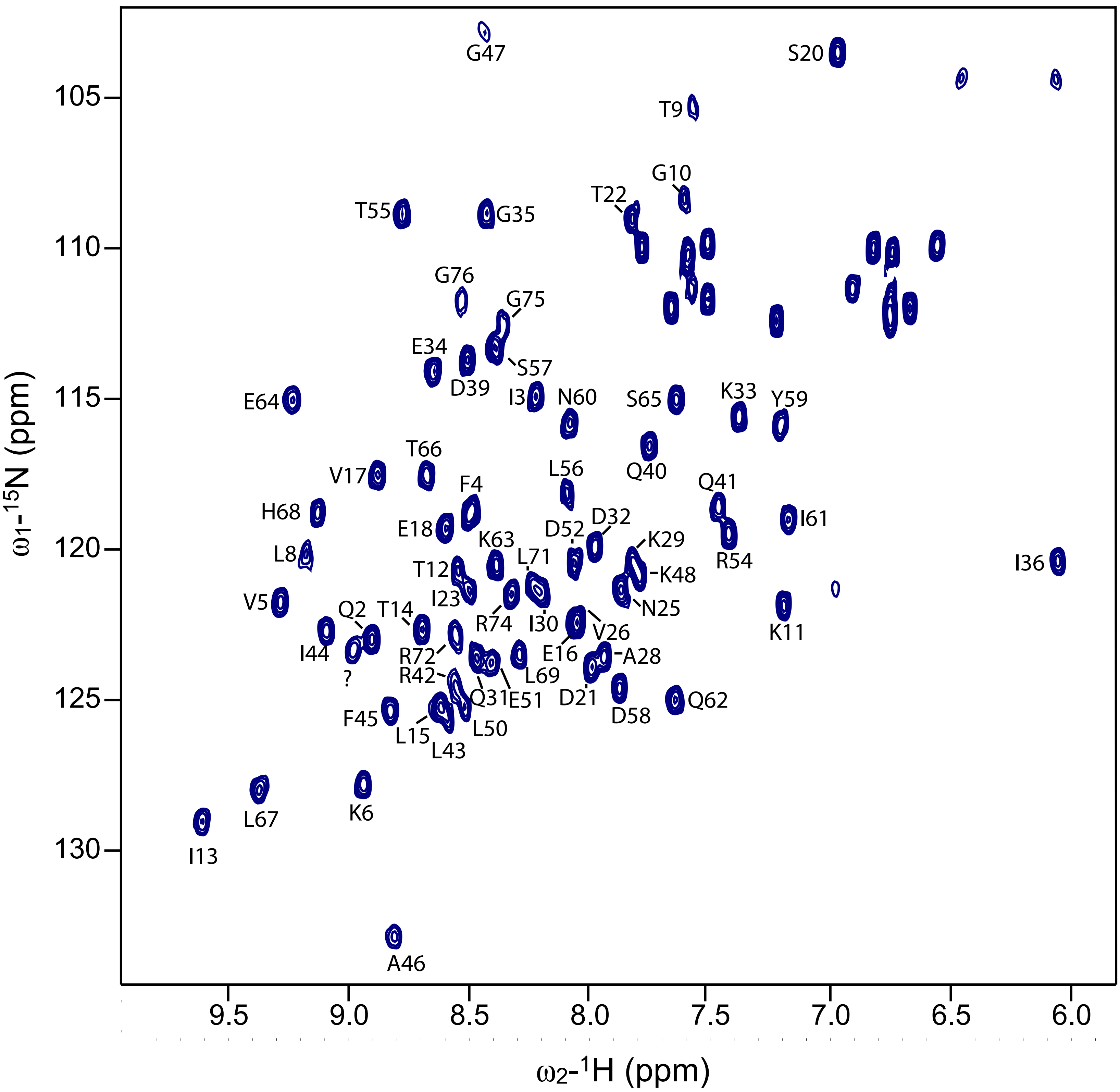

Supplementary Fig. 6 Assignment of ubiquitin charged onto Ube2N. ^{15}N-HSQC spectrum of Ube2N~^{15}N-/^{13}C-Ub. Assignments for amide peaks are given in the spectrum. Unassigned residues are marked by question marks.

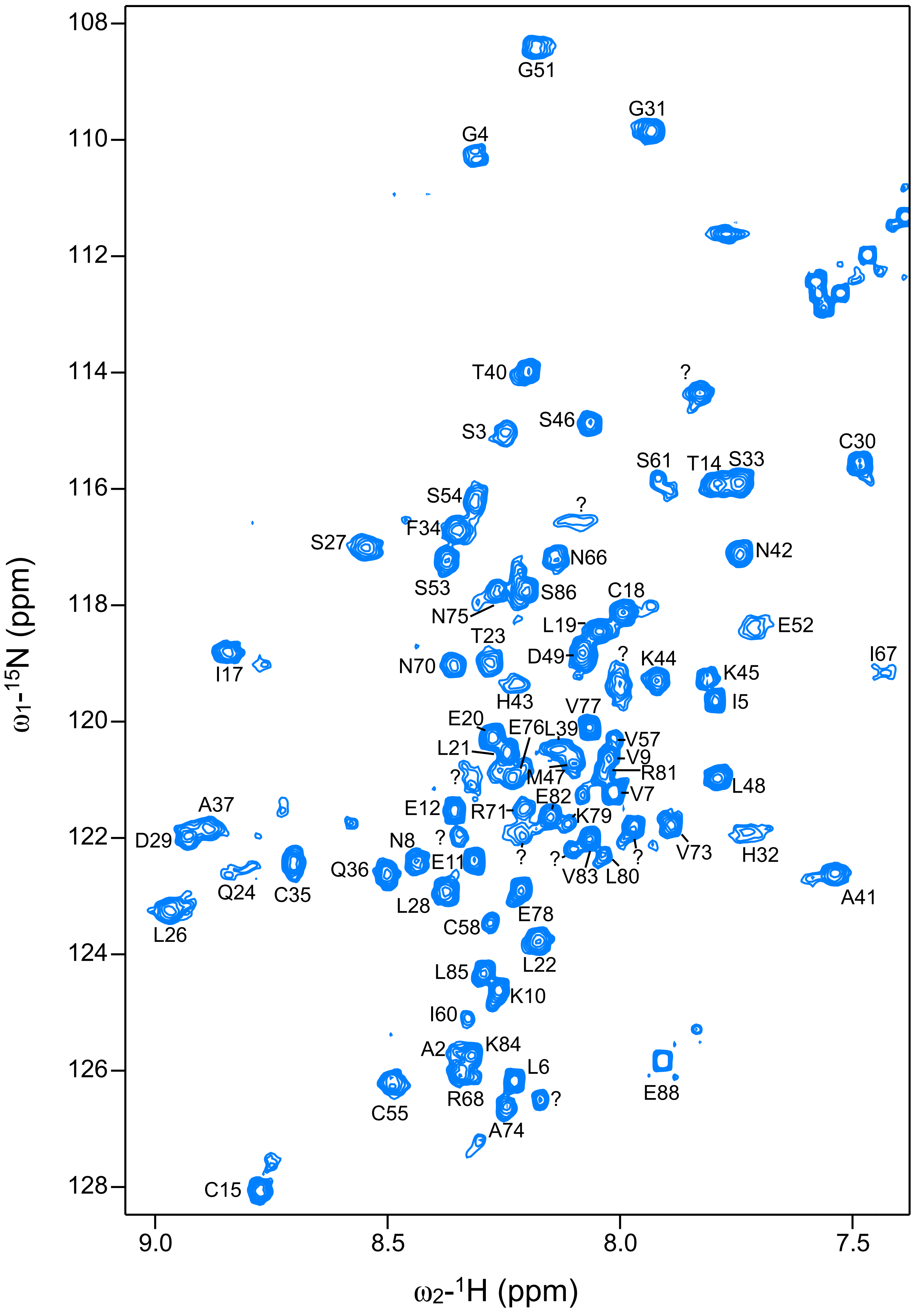

Supplementary Fig. 7 Assignment of TRIM5-RING^{I76E}. ^{15}N-HSQC spectrum of ^{15}N-/^{13}C-T5-R^{I76E}. Assignments for amide peaks are given in the spectrum. Unassigned residues are marked by question marks. The I76E mutation was introduced to remove dimerization of the RING domain, which leads to line broadening.

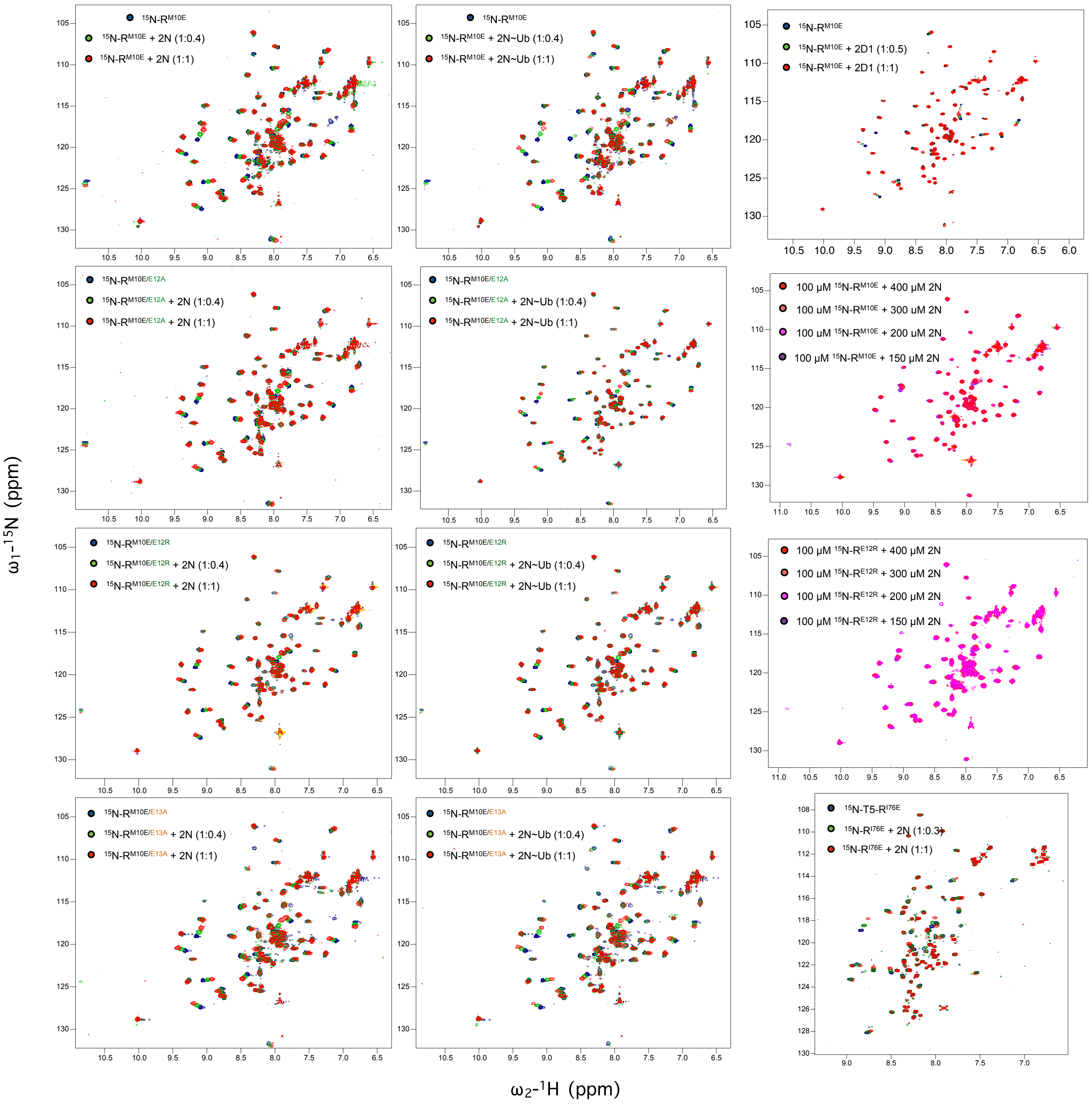

Supplementary Fig. 8 NMR spectra. All the full ^{15}N-HSQC spectra corresponding to results discussed in the text. Constructs and molar ratios are given in the Figure. T21-R^{M10E} constructs are abbreviated as R^{M10E}.

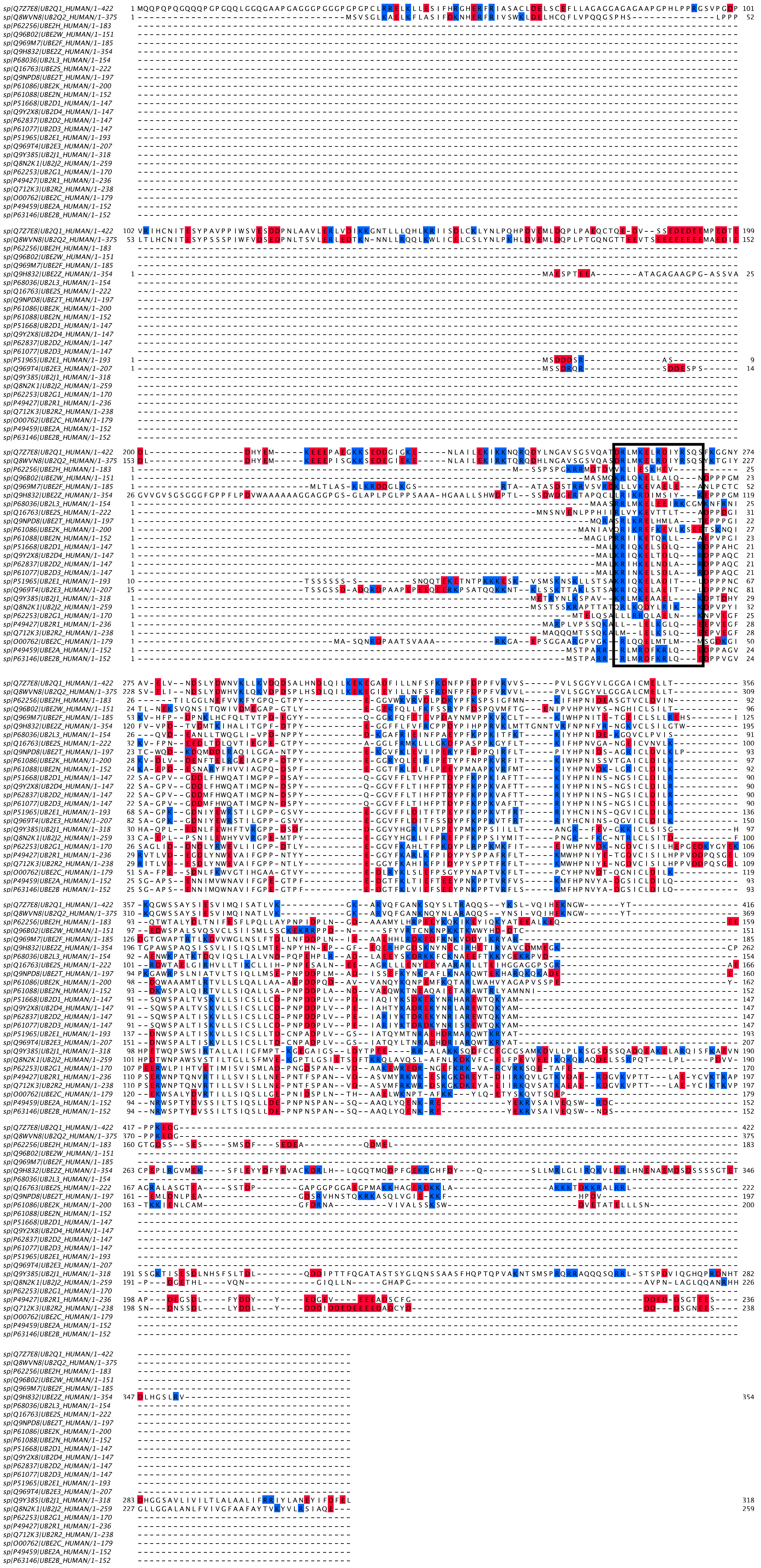

Supplementary Fig. 9 Alignment of E2 enzymes. All E2 enzymes in the biochemical screen in Fig. 1a were aligned using clustal omega.

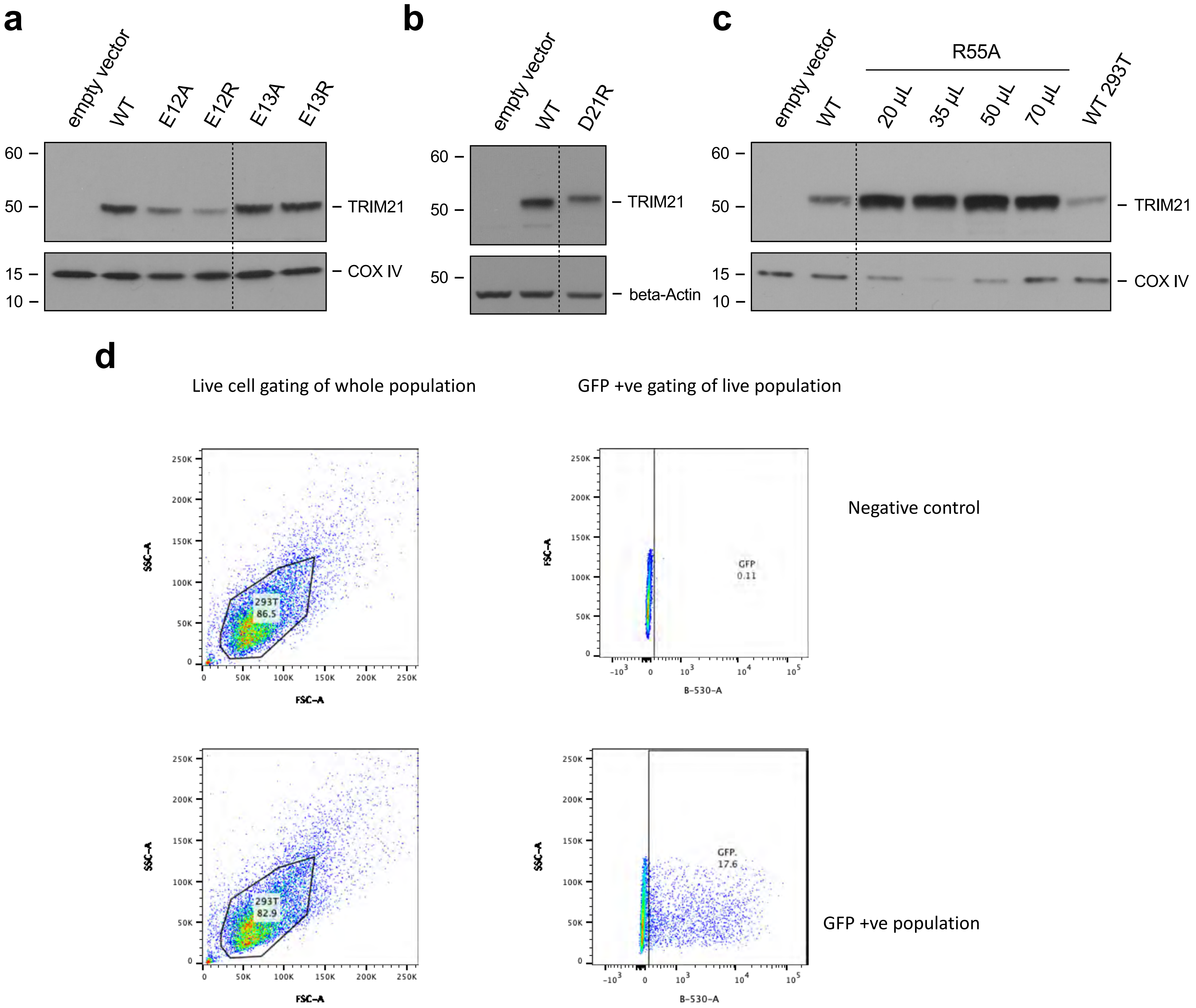

Supplementary Fig. 10 TRIM21 Expression levels. TRIM21 expression level in stably reconstituted 293T cell lines that were used for cell biological assays shown in Fig. 5. **a** Shown are the di-glutamate mutants and **b** D21R. **c** The amount of lentivirus vector containing supernatant was titrated for the *TRIM21*^{R55A} mutants and the cell line that was transduced with 20 µL was used in our assays. The empty vector cells represent TRIM21-KO 293T cells, that were transduced with either WT or TRIM21 mutants. **d** Example contour plots of the gating for live cells and GFP positive cells. Source data are provided as a Source Data file.

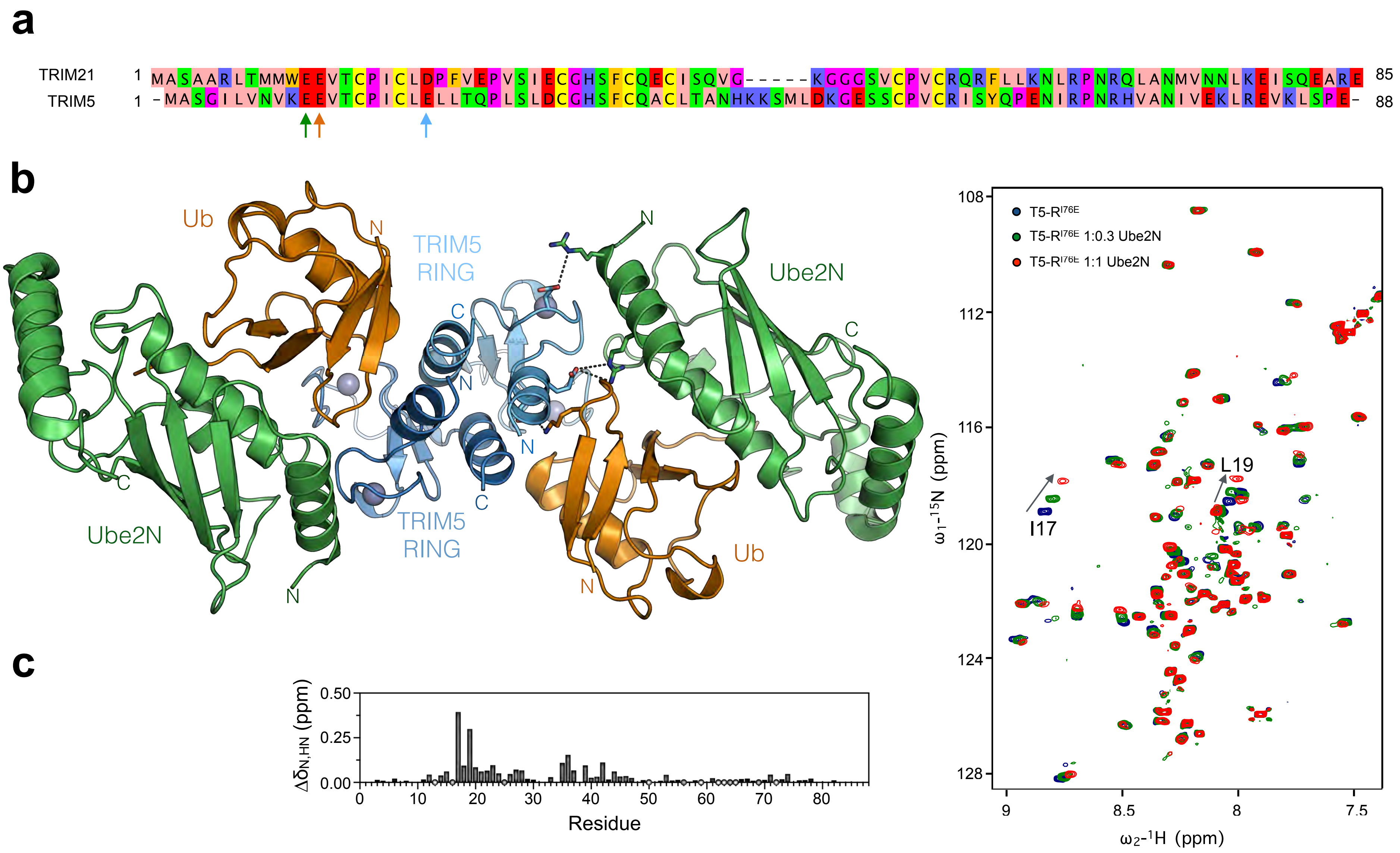

Supplementary Fig. 11 a Sequence alignment of TRIM21 and TRIM5 RING domains. **b** Structural model of TRIM5-RING Ube2N~Ub c omplex based on superposition of the T5-R Ube2N (4TKP)[1] and our T21-R Ube2N~Ub structure. **c** NMR titration spectra of ^{15}N-T5-R^{I76E} with Ube2N. Histogram of the chemical shift perturbation against the T5-R primary structure. Blue circles indicate proline residues, white circles missing assignments. The assigned T5^{I76E} spectrum is shown in Supplementary Fig. 7.

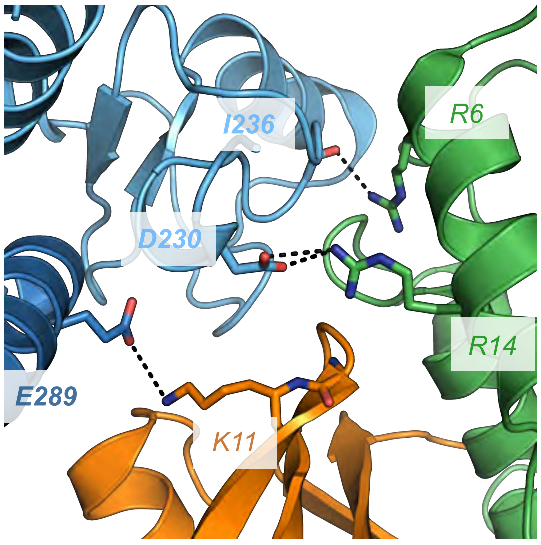

Supplementary Fig. 12 Model of the CHIP$^{U\text{-}box}$:Ube2N~Ub complex. Close-ups of CHIP$^{U\text{-}box}$:Ube2N~Ub model based on the CHIP$^{U\text{-}box}$:Ube2N/Ube2V2 structure (2C2V)[2] and the TRIM21^{RING}:Ube2N~Ub structure presented in this paper, as explained for the TRIM5-RING:Ube2N~Ub model.

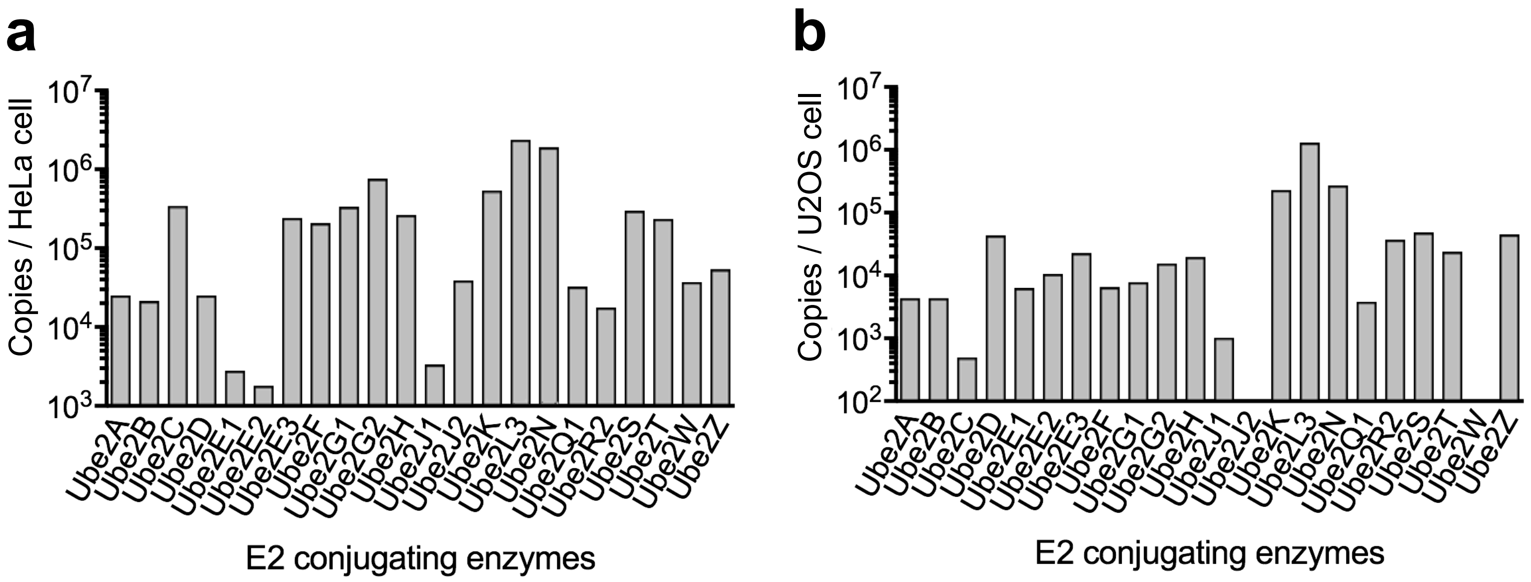

Supplementary Fig. 13 Concentrations of E2 enzymes in cells. Copies of E2 enzymes per cell are shown for **a** HeLa and **b** U2OS cells, as determined by mass spectrometry. Data comes from[3,4], respectively.

Supplementary References

1 Yudina, Z. et al. RING Dimerization Links Higher-Order Assembly of TRIM5alpha to Synthesis of K63-Linked Polyubiquitin. Cell Rep 12, 788-797, doi:10.1016/j.celrep.2015.06.072 (2015).

2 Zhang, M. et al. Chaperoned ubiquitylation--crystal structures of the CHIP U box E3 ubiquitin ligase and a CHIP-Ubc13-Uev1a complex. Mol Cell 20, 525-538, doi:10.1016/j.molcel.2005.09.023 (2005).

3 Beck, M. et al. The quantitative proteome of a human cell line. Mol Syst Biol 7, 549, doi:10.1038/msb.2011.82 (2011).

4 Hein, M. Y. et al. A human interactome in three quantitative dimensions organized by stoichiometries and abundances. Cell 163, 712-723, doi:10.1016/j.cell.2015.09.053 (2015).
