# Supplementary material for: A tri-ionic anchor mechanism drives Ube2N-specific recruitment and K63-chain ubiquitination in TRIM ligases
Source: Nat Commun. 2019 Oct 3;10:4502. doi: 10.1038/s41467-019-12388-y (PMC6776665; doi:10.1038/s41467-019-12388-y)
Supplement: Supplementary file 3 — Reporting Summary [file 41467_2019_12388_MOESM3_ESM.pdf]

nature research

Corresponding author(s): Leo C. James

Last updated by author(s): 2019/08/16

Reporting Summary

Nature Research wishes to improve the reproducibility of the work that we publish. This form provides structure for consistency and transparency in reporting. For further information on Nature Research policies, see Authors & Referees and the Editorial Policy Checklist .

Statistics

For all statistical analyses, confirm that the following items are present in the figure legend, table legend, main text, or Methods section.

n/a	Confirmed	
☐	☒	The exact sample size (n) for each experimental group/condition, given as a discrete number and unit of measurement
☐	☒	A statement on whether measurements were taken from distinct samples or whether the same sample was measured repeatedly
☒	☐	The statistical test(s) used AND whether they are one- or two-sided<br>*Only common tests should be described solely by name; describe more complex techniques in the Methods section.*
☒	☐	A description of all covariates tested
☒	☐	A description of any assumptions or corrections, such as tests of normality and adjustment for multiple comparisons
☒	☐	A full description of the statistical parameters including central tendency (e.g. means) or other basic estimates (e.g. regression coefficient) AND variation (e.g. standard deviation) or associated estimates of uncertainty (e.g. confidence intervals)
☒	☐	For null hypothesis testing, the test statistic (e.g. F, t, r) with confidence intervals, effect sizes, degrees of freedom and P value noted<br>*Give P values as exact values whenever suitable.*
☒	☐	For Bayesian analysis, information on the choice of priors and Markov chain Monte Carlo settings
☒	☐	For hierarchical and complex designs, identification of the appropriate level for tests and full reporting of outcomes
☒	☐	Estimates of effect sizes (e.g. Cohen's d, Pearson's r), indicating how they were calculated

Our web collection on statistics for biologists contains articles on many of the points above.

Software and code

Policy information about availability of computer code

Data collection	Crystallography data collection was performed using setup provided by Diamond Light Source
Data analysis	For Crystallography: XDS, Phenix, Phaser, Phenix_Refine, Coot, Refmac; For NMR: TopSpin, qMDD, CCPN Analysis; For general data visualization: Graphpad Prism7; Pymol, Adobe Illustrator, Apples Keynotes; For Western Blots: Image Studio Lite; For FlowCytometry: FlowJo; for SPR: KaleidaGraph and Graphpad Prism7

For manuscripts utilizing custom algorithms or software that are central to the research but not yet described in published literature, software must be made available to editors/reviewers. We strongly encourage code deposition in a community repository (e.g. GitHub). See the Nature Research guidelines for submitting code & software for further information.

Data

Policy information about availability of data

All manuscripts must include a data availability statement. This statement should provide the following information, where applicable:

- Accession codes, unique identifiers, or web links for publicly available datasets
- A list of figures that have associated raw data
- A description of any restrictions on data availability

PDB code for structure: 6S53. The data availability statement is given in the manuscript.

Field-specific reporting

Please select the one below that is the best fit for your research. If you are not sure, read the appropriate sections before making your selection.

Life sciences study design

All studies must disclose on these points even when the disclosure is negative.

Sample size	Sample size are given in the Figure legend.
Data exclusions	Data exclusion in crystallographic data set was (outer reflection rejection) was carried out automatically as implemented in the program XDS using pre-established criteria. No other data was excluded.
Replication	All attempts at replicates were reproducible. Raw data of replicates is given in Source Data file.
Randomization	We did not randomize any data.
Blinding	No blinding was performed.

Reporting for specific materials, systems and methods

We require information from authors about some types of materials, experimental systems and methods used in many studies. Here, indicate whether each material, system or method listed is relevant to your study. If you are not sure if a list item applies to your research, read the appropriate section before selecting a response.

Materials & experimental systems

n/a	Involved in the study	
☐	☒	Antibodies
☐	☒	Eukaryotic cell lines
☒	☐	Palaeontology
☒	☐	Animals and other organisms
☒	☐	Human research participants
☒	☐	Clinical data

Methods

n/a	Involved in the study	
☒	☐	ChIP-seq
☐	☒	Flow cytometry
☒	☐	MRI-based neuroimaging

Antibodies

Antibodies used	Mouse 9C12 anti-adenovirus 5 hexon IgG was purified from hybridoma obtained from the Developmental Studies Hybridoma Bank, University of Iowa, IA, USA. Humanized anti-adenovirus hexon antibody 9C12 were produced by the Andersen lab for previous studies (Bottermann et al., 2016, Foss et al., 2016). Antibodies used in immunoblots were anti-TRIM21(D-12) Santa Cruz Biotechnology (SC25351), anti-UbcH5 Boston Biochem (A-615), anti-Ube2N Bio-Rad (AHP974), anti-COX IV LI-COR Biosciences (926-42212), anti-Ub-HRP Santa Cruz (sc8017-HRP P4D1), anti-TRIM21 [raised against human TRIM21 RING-B-Box-Coiled Coil (Dickson et al., 2018)], anti-β-actin-HRP Santa Cruz (sc47778). Secondary antibodies were anti-mouse-HRP Sigma (A0168), anti-rabbit-HRP Cell Signaling (7074).
Validation	Specific binding of all antibodies was tested in the laboratory.

Eukaryotic cell lines

Policy information about cell lines

Cell line source(s)	ATCC (293T: CRL-3216; HeLa: CCL-2)
Authentication	Authentication was done by ATCC.
Mycoplasma contamination	All cell lines were tested negative for Mycoplasma contamination.
Commonly misidentified lines (See ICLAC register)	None.

Flow Cytometry

Plots

Confirm that:

- [x] The axis labels state the marker and fluorochrome used (e.g. CD4-FITC).
- [x] The axis scales are clearly visible. Include numbers along axes only for bottom left plot of group (a 'group' is an analysis of identical markers).
- [x] All plots are contour plots with outliers or pseudocolor plots.
- [x] A numerical value for number of cells or percentage (with statistics) is provided.

Methodology

Sample preparation	As stated in Methods under Adenovirus neutralization assay.
Instrument	FACSCalibur, BD Biosciences, San Jose, USA for siRNA experiments and LSRFortessa, BD Biosciences, San Jose, Ca, USA for other virus neutralization experiments.
Software	FlowJo (FlowJo LLC)
Cell population abundance	Stopping gate at 10,000 cells.
Gating strategy	Gating was performed for GFP positive and negative cells. Control were cells that were not infected with GFP labelled virus (background 0.1 %).

- [x] Tick this box to confirm that a figure exemplifying the gating strategy is provided in the Supplementary Information.
